# On the diversity of ideas in academic spaces

**DOI:** 10.3389/fpubh.2023.1301730

**Published:** 2023-11-22

**Authors:** Sandro Galea

**Affiliations:** Office of the Dean, School of Public Health, Boston University, Boston, MA, United States

**Keywords:** universities, free speech, advocacy, public health, Limits

## Abstract

Universities and other academic spaces are social institutions that exist primarily for ideas—to generate, curate, and transmit ideas to students and to the world. Academic spaces cannot carry out that function without supporting a diversity of perspectives. At the same time, universities must reckon—as must all social institutions—with changing norms around how to engage with ideas that challenge, provoke, even anger members of the communities dedicated to their discussion. It is on these institutions to listen to and learn from these ideas, consistent with their core function, while engaging pragmatically with the challenges and controversies that can arise whenever ideas are regularly and freely aired. While there should indeed be limits to the range of tolerable expression within universities, the imposition of these limits should happen rarely, with caution and humility. It is important that academic institutions recognize that while contemporary preoccupations that influence our engagement with speech may change, the secret to universities’ durability over the centuries is that our core purpose—the generation and exploration of ideas—does, and should, not. This inherent value for societies is worth preserving, even, and particularly, in the face of societal pressure and change.

## Introduction

Our social institutions exist as a function of their histories and present social function. For example, we support opera companies both because they preserve and innovate a storied art form and because they continue to deliver ennobling music in the present day. We support museums that preserve creative expressions from history, and also because they continue to educate us about our past, toward a better human future. And yet, these institutions are also, not infrequently, seen at moments in time through the lens of the concerns of the present. Opera companies are, correctly, being challenged for predominantly showcasing the work of white European men, and consequently are being encouraged and pushed to embrace contributions from persons of all identities. The role of museums in high-income countries is being challenged, pushing many museums to ask difficult questions about the provenance of items that they house, and to wonder if those items should be housed in regions where the objects originated. This is the flow of human history and thought. We support institutions that preserve aspects of our past and present that we value—human expression, arts and objects that are worth preserving—but we also expect, correctly, those institutions to be responsive to changes in our thinking about what their role should be, and how we want them to continue to serve our shared humanity in the future. And it is reasonable to expect that institutions that are explicitly supported by the societies in which they are located (at the very least through their largely tax-exempt non-profit status) should function in ways that elevate our ideas about ourselves, to help us all live better.

## The core work of universities and academic spaces

The same is true of universities and academic spaces. I realize that we do not often speak of universities in the same breath as we do opera houses, but universities are also institutions supported by society, aimed largely at preserving historical legacies of thought, and at advancing ideas both through teaching and through scholarship in the present and in the future. The social function of universities is, I would argue—building on much academic work in this space—to develop knowledge, to curate and preserve knowledge, and to transmit that knowledge to society and to students (1–3). While that function can be challenged by the prevailing social ethos, universities lose sight of this core function at their peril. Because we might ask: would society keep supporting the opera if it stopped playing music? Would we keep supporting museums if they moved out of the business of preserving cultural works? I suspect not, and neither would we keep supporting universities if they stopped doing their core work.

Which then brings us to the nature of that work, and how it is best done. How do we best generate knowledge, curate it, and transmit it? There is a broad range of scholarship that has engaged with these questions, tracing the history of universities, and offering perspectives on how universities can best do their work in the present, and I shall not summarize this work here (4–6). However, one core principle that has persevered over time has been the notion that universities should be spaces for the free exchange of ideas, a principle often simply noted as free speech (7, 8). In the public’s mind, and I suspect in many academics’ imagination, universities should be places where anyone can say what they wish to say, when they wish to say it. Tenure, a guarantee of academic appointment given by many globally leading universities, is seen as a protection for faculty to be able to speak the way they wish to speak, free from threat of recrimination by timorous institutions. Speech, the ability to freely think and express one’s thoughts, is in this way regarded as a hallmark of universities, a privilege enjoyed by those fortunate enough to be employed by one such institution, as well as by those in its wider community.

Of course, the story is not that simple. As VanderWeele notes, in both the accompanying piece in this journal, and in a more personal piece previously published elsewhere, there are in the present moment, several forces that curtail the latitude of expression that ostensibly characterizes universities and other academic institutions (9, 10). Central among them is a rigid set of perspectives on what, in a particular field, may constitute acceptable opinions, and a reluctance to allow the expression of differing perspectives within the academy. VanderWeele makes a compelling argument for the importance of such a diversity of perspectives, within universities writ large and within academic public health specifically, and suggests that disagreement that is not disagreeable, that discourse that is civil and respectful should not only be tolerated, but should flourish in universities (9). By and large I agree with these statements. I offer perhaps three additional thoughts, reflecting and building on VanderWeele’s piece, to the end of adding to the idea space that shapes our current reckoning with free expression in universities.

## Reckoning with a range of expression in universities

First, I have little disagreement with the foundational premise that universities are places where ideas should flourish and thrive, and as such that institutions have a responsibility to create an environment where this happens. Building on my earlier comparison, the opera should be a place where musical ideas emerge, and, as a non-musician, I fully expect it is one. It is also clear that ideas, or any creative expression, do not thrive with a preset assumption of what is right and wrong; that our notions of right and wrong, better or worse, are hard won, and therefore we would do well to have the humility to give wide berth to ideas that may seem wrong today, right tomorrow, or were right yesterday, only to be thought wrong today. That means that we should be reluctant to shut down the airing of any idea, and, in fact, we should create a culture where it is understood that ideas are to be tolerated—even if disagreed with strongly. VanderWeele quotes many past thinkers who have reflected this value. I would to that list add Rosa Luxemburg, a key thinker in early twentieth century socialist movements, who noted “Freedom is always and exclusively freedom for the one who thinks differently (11).” We could adopt this in the current context to say that freedom for academic expression only exists if we preserve space for unpopular ideas. Freedom for the expression of ideas that everyone else agrees with is vacuous patting ourselves on the back, and not freedom for anyone. So, the principle that ideas need a context of diversity of thought and perspectives seems to me inviolate, and worth preserving, as I think VanderWeele’s piece argues.

Second, the first point here—an affirmation of the centrality of diverse perspectives within the business of generating new ideas—is almost too easy, too glibly stated, without a serious engagement with what ideas may *not* be tolerated in the academic space. I have written about this previously, and in many ways feel that being clear about what should not be tolerable has an important role in helping us affirm our commitment to everything else we consider to be fair play (12). There is a long jurisprudence, of course, about what speech is not allowable, and similarly a large number of thinkers have engaged with this idea over time (13–16). In the context of the academic environment, I have suggested that we should not tolerate expression that is non-rebuttable, that creates danger for others, that is patently false, and that denies the humanity of others in the academic community (12). I articulate these principles with full awareness that each of them can themselves be the subject of many essays and books, and that their simple expression belies a rather complicated calculus about how best to realize them in practice. So, by way of example, name-calling should not, in my assessment, be tolerated; it is patently non-rebuttable. But when we say danger, do we mean only physical danger? How about psychological danger? Should we limit expression that highlights traumatic experiences (e.g., inter-personal violence) because we know that for persons who have previously experienced traumatic events, a re-experience of these experiences can precipitate anxiety disorders? Perhaps harder yet, what do we say when something being expressed is patently wrong? We are, for example, probably at a point where we would not seriously provide space for a flat-earther on a university campus. More perniciously, neither would we readily tolerate someone who pretends historical catastrophes (e.g., the Holocaust) did not occur. We are also not likely to tolerate someone who espouses beliefs that are simply unmoored to decades of scholarship in a field. For example, it would be hard to see why universities should make space for a public health professor who denies a half-century of research about the carcinogenic effect of smoking. But, on much else, facts are slippery, and one’s notion of what is and is not established fact can differ and change with time. I have previously written about the challenge of establishing the “fact” that lower population levels of salt, for example, may improve population-level health and showed how our understanding of facts around the issue emerge from deeply engrained academic group-think on many sides of the argument (17). And finally, what does denial of humanity mean? Does disagreeing with state-sanctioned same-sex marriage count? These all seem to be positions that require debate and discussion. What is clear is that we should have boundless humility in dealing with these issues, erring on the side of allowing space for all ideas to be heard, recognizing that what we may categorically feel today, may seem like misguided over-reach tomorrow.

Third, I end with a reaction to VanderWeele’s question about how to reconcile the advocacy function of universities with their core functions as discussed here. On this I lean strongly in the direction that we should be exercising an institutional advocacy function sparingly. I have written previously on this, noting “It is only infrequently that we, as a school—as opposed to any of us as individual staff, students, faculty, alums—take a particular position” and suggested what that might look like in practice (18). I also wrote approvingly of former Columbia University President Lee Bollinger’s statement that “[I]t is critically important that the University, as such, not take stands on ideological or political issues. Yet it is also true that the University, as an institution in the society, must step forward to object when policies and state action conflict with its fundamental values, and especially when they bespeak purposes and a mentality that are at odds with our basic mission (18, 19).” These statements suggest that universities and schools can, and should sometimes, lend their voice to advocacy. But this should be done sparingly, and even when it is done, we would do well to remember, as I have written previously, that “it is important to recognize and make space for voices within our community that may not agree with the position we have taken. Just because a position is supported by most of us does not mean it is supported by all of us and those who find themselves in the minority should not fear to speak their mind (18).” So, I fundamentally do not see academic institutions as advocacy institutions, or perhaps any differently than I see the opera which, appropriately, may advocate for music as a centerpiece to our lives, but weigh in sparingly on all other matters. This means that when faculty do weigh in on issues, they have a responsibility to be clear that they are doing so in their own capacity, and in no way representing the institution. It also does not mean, however, that we should not be training students to be activists, recognizing that much social change is accomplished through activism. But academic institutions exist, first and foremost, for the sake of ideas—their generation, curation, transmission. The flourishing of ideas depends on a diversity of ideas, and this diversity is often at odds with institutional advocacy, and the latter, as such, should be applied sparingly indeed. I suppose this means that I disagree with VanderWeele’s note that “Schools of public health…not infrequently also function as public health advocacy organizations (9).” Certainly, if they do, they should not.

## Conclusion

In summary, I agree with the foundational premise that universities should encourage a diversity of perspectives, and that it is the responsibility of universities to create a context which is conducive to this. I simply see no way for universities to carry out their primary social function—the generation, curation, and transmission of ideas—without doing so. I also recognize that the preoccupations of the moment may challenge this goal, in this particular case when our overriding concern with advocacy on deeply felt issues may push us to silence diverging ideas. We should resist this temptation, even as we recognize that there are indeed circumstances when we may wish to curtail expression. Those circumstances, however, should be approached with caution and humility, recognizing that they may change, and that the secret to universities’ durability over the centuries is that our core purpose has changed little, even as contemporary passions have, time and time again.

## Author contributions

SG: Conceptualization, Writing – original draft, Writing – review & editing.
